# The Prevalence of Fabella and Its Association With Osteoarthritic Severity of Knee in Saudi Arabia: A Multicenter Study

**DOI:** 10.7759/cureus.65784

**Published:** 2024-07-30

**Authors:** Lina A Al Mudayris, Abdullah H Alghamdi, Sara Albunyan, Abdulmohsen K Almulhim, Mohammad Alsaleem, Salahulddin Abuljadail

**Affiliations:** 1 Medicine, College of Medicine, King Faisal University, Al-Ahsa, SAU; 2 Orthopedic Surgery, Al Moosa Specialist Hospital, Al-Ahsa, SAU; 3 Orthopedics, King Fahad Hospital Hofuf, Al-Ahsa, SAU; 4 Orthopedic Surgery, College of Medicine, King Faisal University, Al-Ahsa, SAU

**Keywords:** degenerative changes, prevalence, osteoarthritis, knee, fabella

## Abstract

Introduction: The fabella exhibits variable prevalence across populations and is associated with musculoskeletal disorders. Limited research exists on fabella-related studies, particularly in Saudi Arabia, necessitating further investigation to understand its prevalence and association with knee osteoarthritis (OA).

Methods: A retrospective multicenter study was conducted in AlAhsa, Saudi Arabia, reviewing knee X-rays of patients diagnosed with knee OA. Statistical analysis assessed potential associations between fabella presence, demographic factors, and OA severity using the Kellgren-Lawrence classification.

Results: Among 348 participants, 96 (27.6%) exhibited fabella presence, with 38 (39.6%) of them showing fabellar degenerative changes. No significant associations were found between fabella presence and gender, age, knee side, or OA severity, except for a marginal trend in age groups. However, there was an increasing trend in fabellar degeneration with advancing OA grades.

Conclusion: Our study emphasizes the importance of investigating fabella prevalence and its associations with knee OA in diverse populations. While no significant correlations were found in this cohort, the findings prompt further exploration, emphasizing the need for multicenter studies to enhance understanding and clinical management of fabella-related conditions in knee OA.

## Introduction

The fabella is a sesamoid bone that resides in the lateral head of the gastrocnemius posterior to the lateral femoral condyle [1]. The fabella is bound anteriorly by the posterior capsule of the knee joint and posteriorly by the oblique popliteal ligament and lateral gastrocnemius tendon. Moreover, the fabello-fibular ligament extends to its distal insertion at the fibular head [2,3]. The fabella can enhance the effectiveness of the gastrocnemius muscle, reduce friction-induced tendon injury, and cooperate with the fabello-fibular ligament to support the posterolateral aspect of the knee [3-5]. The presence of the fabella is variable, and its reported presence varies across populations. In Western populations, the incidence ranges from 8.7% to 31.3%, while it is higher in Japanese populations, ranging between 66% and 85.8%. Several studies conducted in China have reported a prevalence of fabella ranging from 39.8% to 48.6%. Globally, the fabella is present in approximately 25% of individuals [2-3,6-8]. Fabella is usually a benign structure; however, in some cases, its presence could be associated with musculoskeletal disorders, including chondromalacia fabellae, fabello-femoral osteoarthritis (OA), fabellar dislocation and/or fracture, and fabella syndrome. In rare cases, popliteal artery entrapment syndrome could result secondary to an enlarged fabella [9-15]. Fabella syndrome refers to posterolateral knee pain that is produced by irritation at the posterior side of the lateral femoral condyle and the compression and shearing force between the fabella and lateral femoral condyle [16]. Primary OA is considered the most prevalent arthritic disorder globally, especially in the elderly. Several studies have reported the prevalence of OA in Saudi Arabia. A study done by Al-Arfaj et al. discovered that OA of the knee has a high prevalence in the Saudi population reaching 60.6% in individuals aged 66-75 years [17]. Previous research has demonstrated that individuals with knee OA, the elderly, and those from Eastern countries are more likely to present with fabellae [3,5,18,19]. In this context, there is limited knowledge of fabella-related studies, primarily comprising case reports or studies with small sample sizes. This study aims to investigate the prevalence of fabellae in the Saudi population, considering factors such as gender, age, and knee sides. Additionally, the research aims to analyze the relationships between fabella prevalence or degeneration and age, as well as their potential associations with knee OA in multiple centers in Al-Ahsa, Saudi Arabia.

## Materials and methods

Study design

This retrospective study was approved by the local ethics committee (Ethical approval number: No. 17-EP-2024). A retrospective review of patients who underwent X-ray in Al-Ahsa hospitals including King Fahad Hospital Hofuf, Prince Saud Bin Jalawi Hospital, and Maternity Children Hospital from September 1st to November 31st in 2023 was conducted. Patients with non-weight-bearing anteroposterior and lateral radiographs showing OA were included. Exclusion criteria included: (a) no signs of joint degeneration; (b) joint replacement or fracture; (c) inadequate X-ray, lacking anteroposterior and lateral X-rays; and (d) poor image quality. The data for the study was collected by trained medical students who conducted a review of X-rays obtained from the Picture Archiving and Communication System (PACS). A total of 740 X-rays were taken from the database. However, 392 (53.0%) of these were not included in the analysis as they did not meet the inclusion criteria. Thus 348 participants (47.0%) were included in the final analysis (198 females (56.9%) and 150 males (43.1%)), aged 16-91 years.

Radiographic scorings of knee osteoarthritis and fabella degeneration

In our study, the Kellgren-Lawrence classification system was utilized to categorize patients with knee OA into five distinct grades: Grade 0 (none), indicating a clear absence of x-ray indicators of OA; Grade 1 (doubtful), indicating uncertain joint space narrowing and potential osteophytic lipping; Grade 2 (minimal), indicating the presence of definite osteophytes and possible joint space narrowing; Grade 3 (moderate), indicating moderate presence of multiple osteophytes, clear joint space narrowing, some sclerosis, and potential bone end deformity; and Grade 4 (severe), indicating large osteophytes, significant joint space narrowing, severe sclerosis, and definite bone end deformity [20]. The x-rays were evaluated for the presence of the fabella and for any degenerative changes. The fabella was classified into two categories: showing degenerative changes, indicated by sharp, irregular edges; and showing no degenerative changes, characterized by a smooth and round appearance.

Statistical analysis

Both descriptive and inferential statistical analysis of the data was carried out. Simple descriptive statistics of the sociodemographic characteristics and other categorical variables in the form of frequencies and percentages were calculated and tabulated. For continuous variables, means and standard deviations were reported as measures of central tendency and dispersion, respectively, owing to the large sample size and relatively normal distribution of the variables assessed by Q-Q plots. Additionally, the data was visualized where possible for easier interpretation.

In order to find out the factors associated with the prevalence of the fabella, Fischer’s exact test was applied and interpreted for categorical variables and independent samples t-test for continuous variables. Significance was established at a p-value of 0.05 or less indicating a 95% confidence interval. All statistical calculations were performed using IBM SPSS version 27.0.1 (IBM Corp., Armonk, NY).

## Results

**Figure 1:**
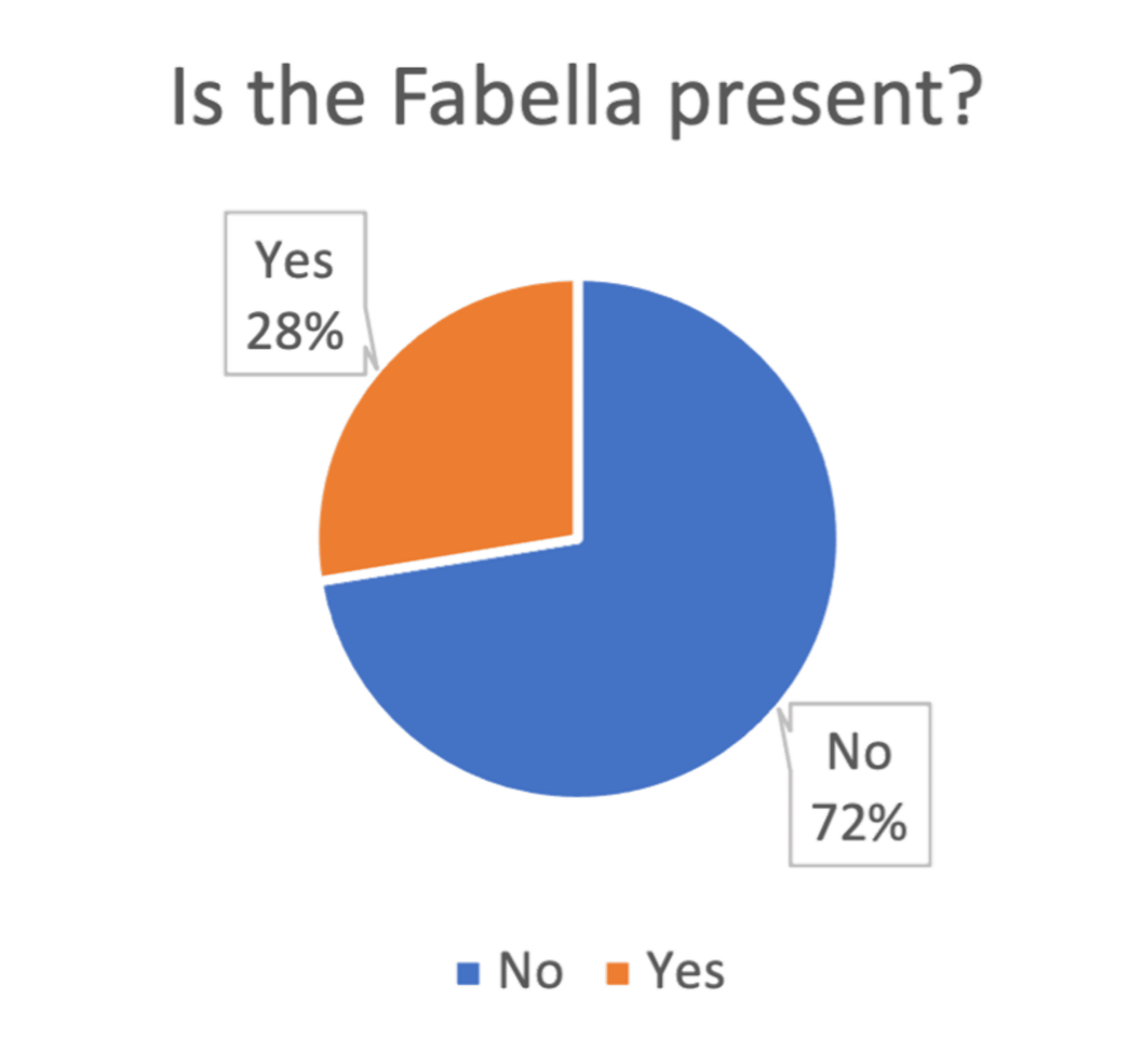
Prevalence of fabella among the participants

**Figure 2:**
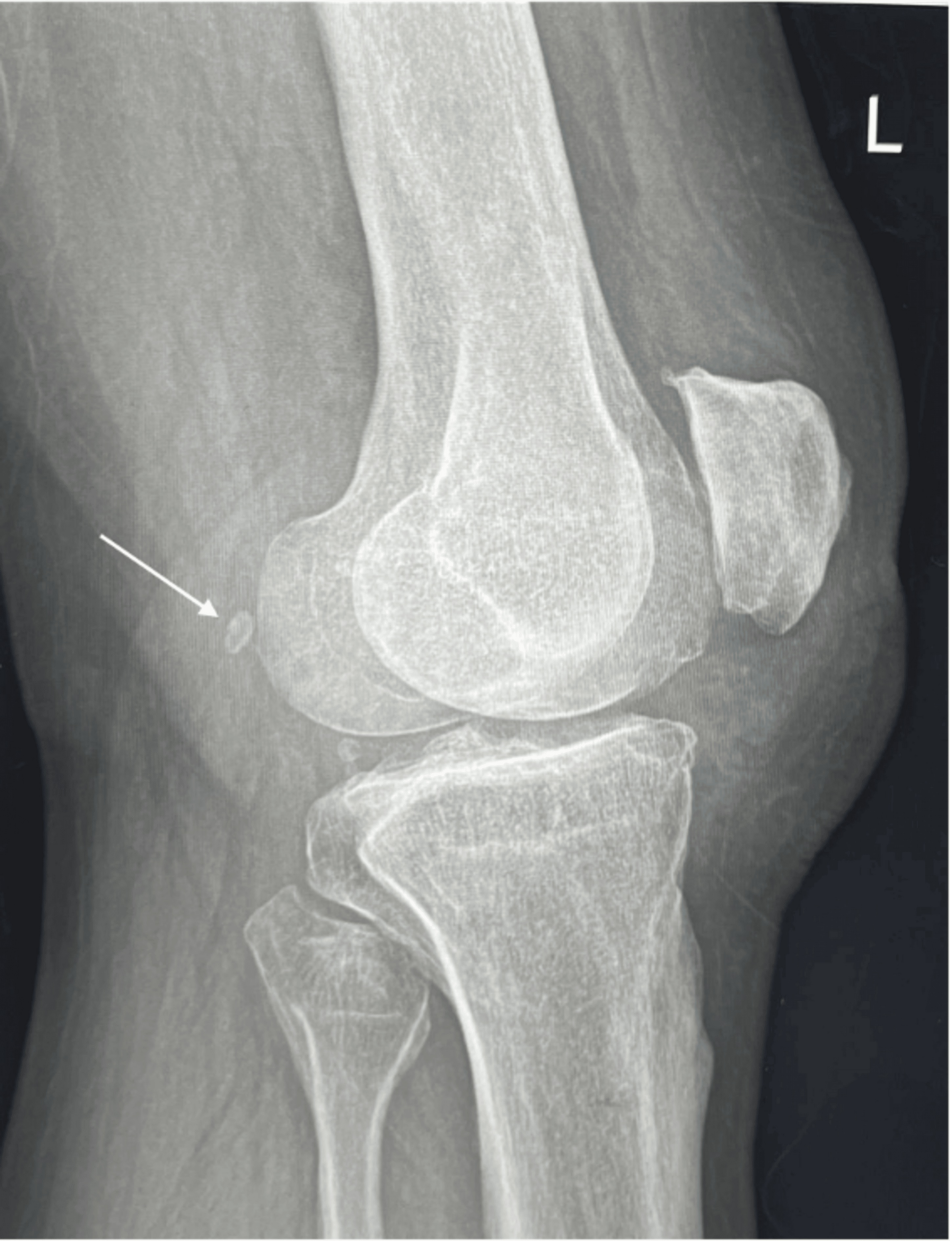
Lateral view of knee joint showing degenerative changes of fabella (white arrow) along with knee OA OA, osteoarthritis

A total of 348 X-rays met our inclusion criteria. In this cohort of 348 participants (198 (56.9%) female and 150 (43.1%) male) aged 16 to 91 years (mean age: 52.7±13.2), the distribution across age categories revealed a predominant representation between 41 to 60 years (41-50: 105 (30.2%) and 51-60: 97, 153 (27.9%)). The majority underwent X-rays for the left knee 195 (56.0%) compared to the right knee (44.0%). Assessment of OA grade using the Kellgren-Lawrence classification showed a relatively even distribution across grades one to four, with 101 (29.0%), 109 (31.3%), 103 (29.6%), and 35 (10.1%), respectively. Among these participants, 96 (27.6%) exhibited the presence of a fabella, while 252 (72.4%) did not (Figure 1). Of those with a fabella, 38 (39.6%) displayed degenerative changes (Figure 2), whereas 58 (60.4%) had a smooth, round morphology of the fabella, indicating no degenerative alterations (Table 1).

**Table 1:** Sociodemographic and clinical characteristics of the participants (n=348) OA, osteoarthritis

					N	%
Age	M	SD				
	52.7	13.2				
Gender				Male	150	43.1%
				Female	198	56.9%
Age categories				11-20	6	1.7%
				21-30	8	2.3%
				31-40	41	11.8%
				41-50	105	30.2%
				51-60	97	27.9%
				61-70	56	16.1%
				71-80	28	8.0%
				81-90	5	1.4%
				91	2	0.6%
Knee				Left	195	56.0%
				Right	153	44.0%
OA grade (Kellgren-Lawrence)				1	101	29.0%
				2	109	31.3%
				3	103	29.6%
				4	35	10.1%
Is the fabella present?				No	252	72.4%
				Yes	96	27.6%
Are there any degenerative changes to the fabella? (N=96)				No	58	60.4%
				Yes	38	39.6%

Factors associated with prevalence of fabella

**Figure 3:**
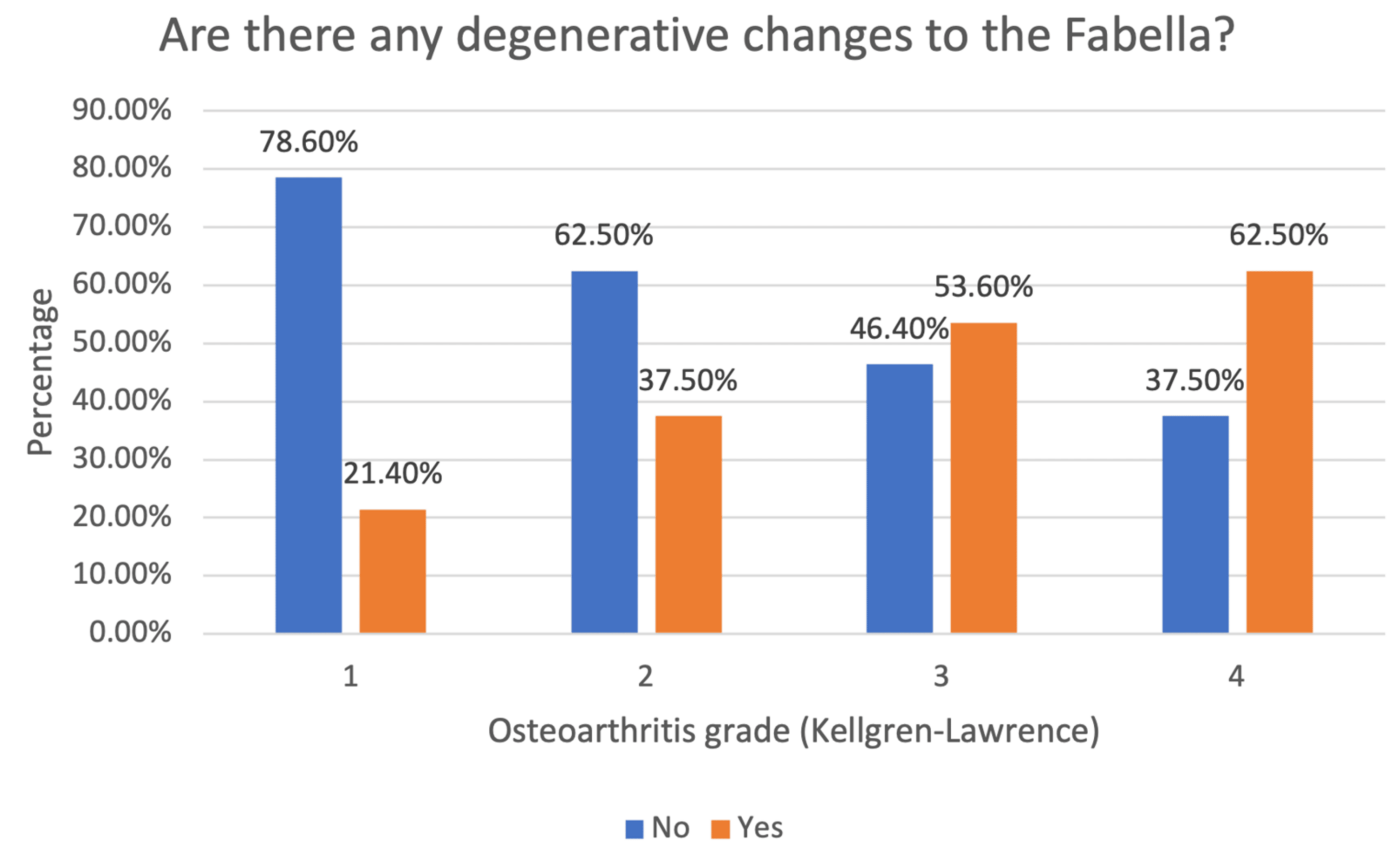
Association of degenerative changes to the fabella with OA grade (n=96) OA, osteoarthritis

The investigation aimed to discern potential associations between fabella prevalence and various parameters among 348 participants. Analysis showed no significant link between gender and fabella presence (p=0.398). Notably, 147 (74.2%) of females and 105 (70.0%) of males lacked the presence of fabella, indicating a comparable distribution between genders. Similarly, no substantial association was evident between OA grades (Kellgren-Lawrence) and fabella presence (p=0.920), with proportions ranging from 70.6% to 77.1% across different grades without significant deviation. While a trend emerged in the age groups (p=0.053), it did not reach significance. Notably, the youngest group (16-20 years) exhibited no fabella presence, whereas the oldest (91-100 years) showed 100% prevalence. Concerning the knee side, no significant association with fabella presence was observed (p=0.257), with proportions around 70-75% lacking the fabella on either side. These findings indicate a lack of substantial correlation between gender, OA grade, knee side, and fabella presence, with only a marginal trend noted in the age groups (Table 2). The research findings reveal an increasing trend in the percentage of degenerative changes to the fabella with advancing OA grades, with Grade 1 exhibiting the lowest proportion of six (21.40%) and Grade 4 demonstrating a comparatively highest percentage of five (62.50%) (Figure 3).

**Table 2:** Association of prevalence of fabella with gender, age groups, OA grade, or side of the knee (n=348) OA, osteoarthritis

		Is the fabella present?				
		No		Yes		P-value^F^
		N	Row %	N	Row %	
Gender	Female	147	74.2%	51	25.8%	0.398
	Male	105	70.0%	45	30.0%	
Age groups	11 - 20	6	100.0%	0	0.0%	0.053
	21 - 30	3	37.5%	5	62.5%	
	31 - 40	34	82.9%	7	17.1%	
	41 - 50	75	71.4%	30	28.6%	
	51 - 60	73	75.3%	24	24.7%	
	61 - 70	38	67.9%	18	32.1%	
	71 - 80	19	67.9%	9	32.1%	
	81 - 90	4	80.0%	1	20.0%	
	91	0	0.0%	2	100.0%	
OA grade (Kellgren-Lawrence):	1	73	72.3%	28	27.7%	0.920
	2	77	70.6%	32	29.4%	
	3	75	72.8%	28	27.2%	
	4	27	77.1%	8	22.9%	
Knee	Left	138	70.8%	57	29.2%	0.257
	Right	114	74.5%	39	25.5%	
^F^Fischer’s exact test						

## Discussion

This study investigated the prevalence of the fabella and its association with the osteoarthritic severity of the knee joint using the Kellgren-Lawrence scale in a diverse cohort of 348 participants in Saudi Arabia using conventional radiography. In this cohort, the prevalence of the fabella in participants who were diagnosed with OA was 96 (27.6%). Information from epidemiological investigations conducted in diverse regions globally has suggested considerable variability in the prevalence of the fabella across distinct ethnic populations [21]. A recent systematic review encompassing 86 studies conducted from 1975 to 2020 estimated that the prevalence of fabella globally is 25% in the general population and rises to 51% among individuals with OA [7]. Another systematic review conducted in Korea determined the prevalence of fabella in the Korean population and explored potential changes in prevalence over time revealing that 52.83% of individuals exhibited the presence of fabellae [22]. Moreover, research conducted by Zhong et al. in China examined the prevalence of the fabella retrospectively and found that the overall occurrence of the fabella was 39.8%, involving 402 out of 1011 knees [8].

In this study, the lack of a significant correlation between gender and the presence of the fabella (p=0.398) aligns with the findings of various studies, indicating an equal likelihood of both men and women having a fabella [22,23-25].

The analysis of demographic factors revealed that age, gender, and knee side did not exhibit substantial correlations with fabella presence, except for a marginal trend observed in the age groups. Despite a slightly higher mean age in participants with a fabella, this difference was not statistically significant (p=0.197), suggesting that age may not be a decisive factor in determining fabella presence in this cohort. In line with our findings, Tabira Y et al. found that age is not correlated to the frequency of fabella [26]. Nonetheless, several studies observed that the overall occurrence of the fabella rose with advancing age [21,7-8,23,27,28].

There was no pattern recognized regarding the affected knee side leading to a negligible association between the knee side and the presence of the fabella (p=0.257). This could be attributed to the unilateral review of knee X-rays in our study since the assessment of the fabella's presence does not necessitate bilateral knee X-rays. Nevertheless, multiple studies demonstrated that the occurrence of fabellae on both sides, affecting both males and females, was twice as common as the presence of fabellae on only one side, with a ratio of 2:1 [25,18,29].

It is thought that as the severity of OA increases, the likelihood of the presence of the fabella also increases [28]. In this study, the severity of OA was assessed using the Kellgren-Lawrence scale. In the current study, 28 (27.7%) subjects with OA Grade 1 manifested the presence of fabella while for subjects with OA Grades 2,3 and 4, the prevalences were 32 (29.4%), 28 (27.2%), and eight (22.9%), respectively, resulting in no considerable association between OA grades and the fabella presence (p=0.920). Interestingly, this finding contrasts with other papers that have identified a significant association between OA severity and the presence of the fabella.

Among the study participants with fabella, 38 (39.6%) subjects presented with degenerative changes to their fabella. These degenerative changes are thought to be correlated to the degree of OA calculated by the Kellgren-Lawrence scale. The presence of fabellar degenerative changes is more probable in cases of severe OA [8]. Out of the eight subjects with OA Grade 4, five (62.5%) displayed degenerative changes to their fabella. Consistent with our findings, a study conducted in China reported a positive correlation between degeneration levels of fabellae and the grades of OA severity within the corresponding knee [30].

The variance between the outcomes of this study and other research may be attributed to the inherent subjectivity of the Kellgren-Lawrence scale. The impact of personal bias can result in diverse evaluations of severity and prevalence in group studies. It is recommended that, whenever feasible, all population studies aiming for comparisons should ensure that X-rays are evaluated by the same observer or, preferably, by two observers in consultation [20]. This approach can enhance the uniformity and reliability of assessments, mitigating potential biases in the interpretation of both OA severity and fabella prevalence.

The current study acknowledges certain limitations, with one notable concern being its limited study location. The research focused solely on the Al-Ahasa region, neglecting the inclusion of other regions across Saudi Arabia. This restricted geographic scope might impact the generalizability of the findings to the entire Saudi Arabian population, as regional variations could exist. Our study recognizes that knee joint X-rays were not reviewed bilaterally for each participant, as not all participants underwent X-rays for both knees. Consequently, the study might not have captured a comprehensive picture of the bilateral presence or absence of the fabella. This limitation introduces a potential source of bias, as the absence of bilateral X-ray assessments restricts our ability to accurately identify consistent patterns or associations between the affected knee side and the presence of the fabella. Therefore, the reported negligible association may be influenced by the incomplete data on bilateral knee evaluations. To enhance the robustness and reliability of future research, it is advisable to undertake multicenter studies that involve a more extensive and diverse participant pool. This approach would not only address the regional limitation but also contribute to a more comprehensive understanding of the prevalence and associations of the fabella across different demographics in Saudi Arabia. Moreover, it is advisable to develop and implement a more objective classification system for knee OA in future studies. This proactive measure will minimize potential sources of bias and mitigate the risk of misinterpretation in assessing both OA severity and fabella prevalence.

## Conclusions

In a 27.6% fabellar, occurrence was observed among participants with OA. While no significant correlations were found between fabella presence and age, gender, or knee side, a marginal trend in age groups hints at potential influences. Despite limitations, the findings prompt further exploration, suggesting a complex relationship between the fabella and knee OA that challenges existing perspectives. Future multicenter studies, encompassing diverse populations, are recommended to enhance the generalizability of findings and delve deeper into fabellar prevalence and its associations in Saudi Arabia. Additionally, exploring correlations between fabellar degeneration and OA severity warrants attention in future research.
